# Application of color Doppler ultrasound combined with Doppler imaging artifacts in the diagnosis and estimate of congenital hypertrophic pyloric stenosis

**DOI:** 10.1038/s41598-017-10264-7

**Published:** 2017-08-25

**Authors:** Suihong Ma, Jianhua Liu, Youxiang Zhang, Yuwen Yang, Hai Jin, Xiaomei Ma, Hongqin Wei

**Affiliations:** 1 The First Affiliated Hospital of Jinan University, Guangzhou, China; 2 Department of Medical Ultrasound, Guangzhou First People’s Hospital, Guangzhou Medical University, Guangzhou, China; 3 The Second Affiliated Hospital of South China University of Technology, Guangzhou, China; 4 Department of Pediatrics, Guangzhou First People’s Hospital, Guangzhou Medical University, Guangzhou, China; 5 Department of Medical ultrasound, BaoAncentral Hospital of Shenzhen, Guangdong, China

## Abstract

Congenital Hypertrophic Pyloric Stenosis (CHPS) is a disease condition that is caused as a result of pylorus wall hypertrophy and hyperplasia. In this study, we used color Doppler flow imaging (CDFI) and Doppler artifacts technique to observe the blood flow of hypertrophic pylorus tissue and the dynamic imaging of liquid passing through the pyloric canal in CHPS infants. 65 cases of CHPS infants and 50 infants without CHPS served as control group. We found that there were statistically significant differences between the blood flow grade of muscular layer and mucosal layer between CHPS and control infants, but no significant differences were observed in the same group. Doppler artifacts technique demonstrated the whole process of contrast agent flow through pyloric canal was directly observed in 35 of 65 subjects, and the internal diameter of the pyloric canal was 1.93 ± 0.33mm.Conclusion that CDFI combined with color Doppler artifacts technique was proved to be effective to observe the distribution feature and blood flow grade in each layer of pyloric canal in CHPS patients. This method provides the evidence for judging the degree of pyloric stenosis clinically, and furnishes the basis of therapy along with its clinical significance and good application value.

## Introduction

Congenital Hypertrophic Pyloric Stenosis (CHPS) is a disease condition that is caused as a result of pylorus wall hypertrophy and hyperplasia, and incomplete mechanical obstruction. This has been the most common etiology of vomiting in infants^1^. The incidence rate was approximately 2 to 5 per 1000 live births, with infants of affected parents at greater risk (2.5–20%). The risk is four times higher in boys than girls^2, 3^. The pyloric hypertrophies after birth causes progressive gastric outlet obstruction and most commonly observed in infants between 2 and 6 weeks of age^4^. The main clinical symptoms include frequent vomiting, usually 10 to 30 min after feeding, palpable in the middle of the abdomen and olive-shaped lumps, as well as feeding visible peristaltic waves^5^. For a better prognosis results, early diagnosis and early treatment are required. If it is not timely diagnosed and treated, it causes severe malnutrition and death^6^. The disease can be diagnosed mainly by imaging examination, the upper gastroenterography being the most successful with the longest history^7^. However, upper gastroenterography cannot directly display the hypertrophic pyloric and the damage caused by the gastroenterography to the baby cannot be ignored, the barium and aspiration risk^8, 9^.

In recent years, with the growing popularity of ultrasound and the development of ultrasonic technology, it directly reflects the situation of Helicobacter pylori, easy to measure the thickness of the muscle and the length of the tube, and has no damage, safe, simple and can be easily performed repeatedly. Therefore, at present, ultrasound examination has been an important screening tool for most of the children with clinical CHPS^10–12^.

Color Doppler flow imaging (CDFI) is a technique for examining blood flow using Doppler’s principle. Compared to other techniques, CDFI provides a noninvasive, real-time information flow signal of the lesion area. In the color sampling frame of CDFI, the color signal of the non-real blood flow is color Doppler artifacts image^13^. At present, the international literatures on ultrasonic examination of CHPS are mostly two-dimensional ultrasound images, few literatures reported that CDFI was used to analyze the blood flow signals of hypertrophic pyloric in CHPS^14–16^. In this study, the blood flow distribution characteristics of each layer of the mucosal layer during CHPS were observed and examined by CDFI, and provided objective information clinically. This helped the doctors to prevent the losses of large blood vessel during surgery, and in turn reduces the risk of surgery. Hemanz-schulman^17^ in his study graded the blood flow of pyloric hypertrophy and in this paper, we used this method to evaluate the abundant blood supply of pyloric hypertrophy, which also provides effective information clinically. At the same time we used color Doppler artifacts technology, which is more intuitive and dynamic to observe the whole process of liquid passage through the pyloric canal. Similarly, this in turn can make up with the two-dimensional ultrasound conditions for liquid echo and pyloric canal echo contrast which is not strong and can be easily missed. The purpose of this paper is to discuss how the application of CDFI combined artifacts imaging technique innovatively in the diagnosis of CHPS, and provide more objective basis for clinical evaluation of pyloric stenosis.

## Results

### Clinical manifestations between CHPS and control groups

There were 59 males and 6 females, aged 15 to 103 days in the CHPS group, with male prevalence ratio of 9.8:1,with a mean age of 42.48 days ±17.21 (SD), a mean weight of 3.77 kg ± 0.56 (SD). And there were 27 males and 23 females, aged 20 to 88 days in the control group, with male prevalence ratio of 1.2:1, with a mean age of 41.58 days ± 12.70 (SD), a mean weight of 4.90 kg ± 1.18 (SD) (Table 1). Compared with the control group (N = 50) infants who did not have CHPS, the differences in male prevalence ratio and the mean weight were significant.

**Table 1:** Clinical manifestations in the studied groups of infants.

Groups of patients	No. of patients	M	F	M/F rate	Age(days), median(range)	Weight(kg), median(range)
CHPS	65	59	6	9.8: 1	38.58 ± 15.32 (15–103)	3.43 ± 0.60 (2.2–6.6)
Control	50	27	23	1.2: 1	41.58 ± 12.70 (20–88)	4.90 ± 1.18 (3.7–10.8)

M – male, F – female; CHPS –congenital hypertrophic pyloric stenosis.

### Comparison of pyloric parameters between CHPS and control group

CHPS was present in 65 infants, who satisfied the ultrasound diagnostic criteria, the characteristics of the ultrasound showed that the longitudinal section of the pyloric canal was like a cervix, and the transverse section was like a target ring. Compared with the control group, the differences in muscular layer thickness, the length of pyloric canal and the diameter of pylorus were highly significant (P = 7.44E-44, 3.01E-73, 5.77E-29), and the thickness of mucosal layer remained significant (P = 6.34E-18) (Table 2).

**Table 2:** 65 cases of CHPS group compared with the ultrasonic measurement of 50 cases of control group (mm).

Group	Control	CHPS	P value
Muscular layer	2.02 ± 0.45	4.85 ± 0.82	7.44E-44
Mucosal layer	0.74 ± 0.28	1.24 ± 0.18	6.34E-18
Length of pylorus	9.68 ± 0.25	18.53 ± 0.91	3.01E-73
Canal diameter of pylorus	3.42 ± 0.52	1.95 ± 0.35	5.77E-29

### The distribution feature of blood flow in the CHPS group

**Figure 1:**
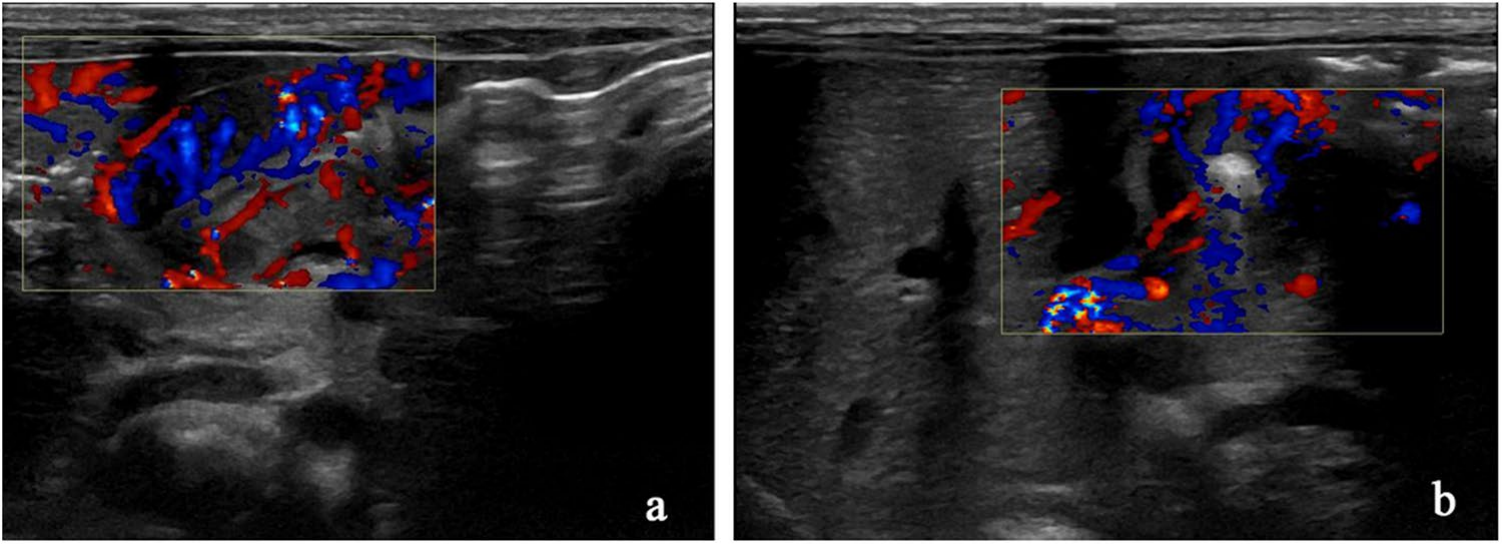
The distribution feature of blood flow in the CHPS group. (**a**) On the longitudinal section of pyloric showed muscle was thick and short rod arranged in parallel, the serosal layer and mucosal layer was continuous strip; (**b**) On the transverse section of pyloric showed muscle was radiating arranged, the blood flow in the serosal layer and mucosal layer cannot be displayed.

The distribution feature of blood flow in each layer of pyloric canal mucosa was observed clearly by CDFI examination in the CHPS group: Longitudinal section of pylorus showed that the blood flow in the pyloric muscle was thick and a short rod was arranged in parallel and perpendicular to the pyloric diameter, the blood flow in the serosal layer and mucosal layer was continuously stripped and was parallel to the pyloric canal diameter (Fig. 1a). On the transverse section of pylorus showed that the blood flow in the pyloric muscle was radiated and the blood flow direction was annular blood flow, but some segments could not be displayed because of parallel arrangement with sound beam (Fig. 1b). In the control group the pyloric canal mucosa of each layer was small with poor blood flow.

### The difference of the grade of flow between CHPS and control group

In the control group, the mean grade of flow in the muscular layer was 1.58 ± 0.57 and in the mucosal layer 1.42 ± 1.50. In the CHPS group, the mean grade of flow in the muscular layer was 2.83 ± 0.38 and in the mucosal layer 2.91 ± 0.30. Among them, we identified 11 infants whose inflow was grade II and 54 infants inflow was grade III in the muscular layer, 6 infants showed an inflow of grade II and 59 infants with an inflow of grade III in the mucosal layer. The differences in the muscle and mucosal flow grade between the CHPS and control groups was highly significant (*t value is 13*.*33*, *18*.*77*, *both p* < *0*.*01*, Table 3).There was no difference observed in the blood flow between muscle and mucosal flow grade in both control (*t value is 1*.*49*, *p* > *0*.*05*) as well as CHPS group (*t value is 1*.*30*, *p* > *0*.*05*).

**Table 3:** The difference of the grade of flow between CHPS and control group.

Group	Control	CHPS	t value	P value
Mean grade of flow in the muscular layer	1.58 ± 0.57	2.83 ± 0.38	13.33	2.52E-22
Mean grade of flow in the mucosal layer	1.42 ± 0.50	2.91 ± 0.29	18.77	3.66E-30
t value	1.49	1.30		
P value	0.07	0.10		

### Time and frequency of liquid passage through the pyloric canal and the pyloric diameter

**Figure 2:**
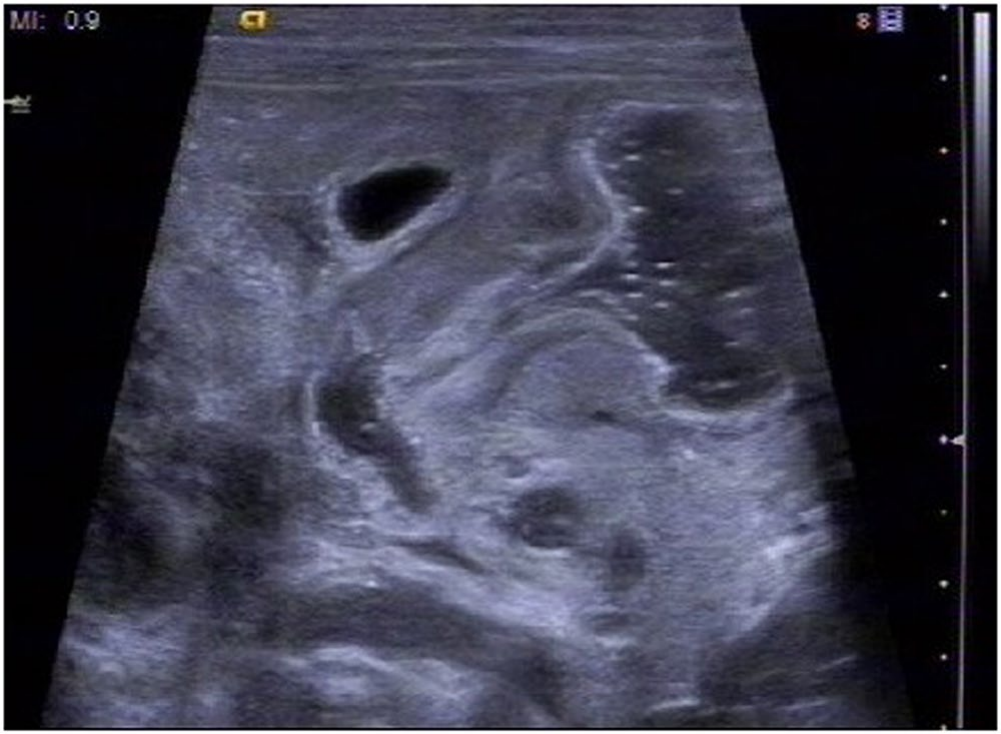
Two dimensional ultrasound for examining the liquid passing through the Pylorus.

**Figure 3:**
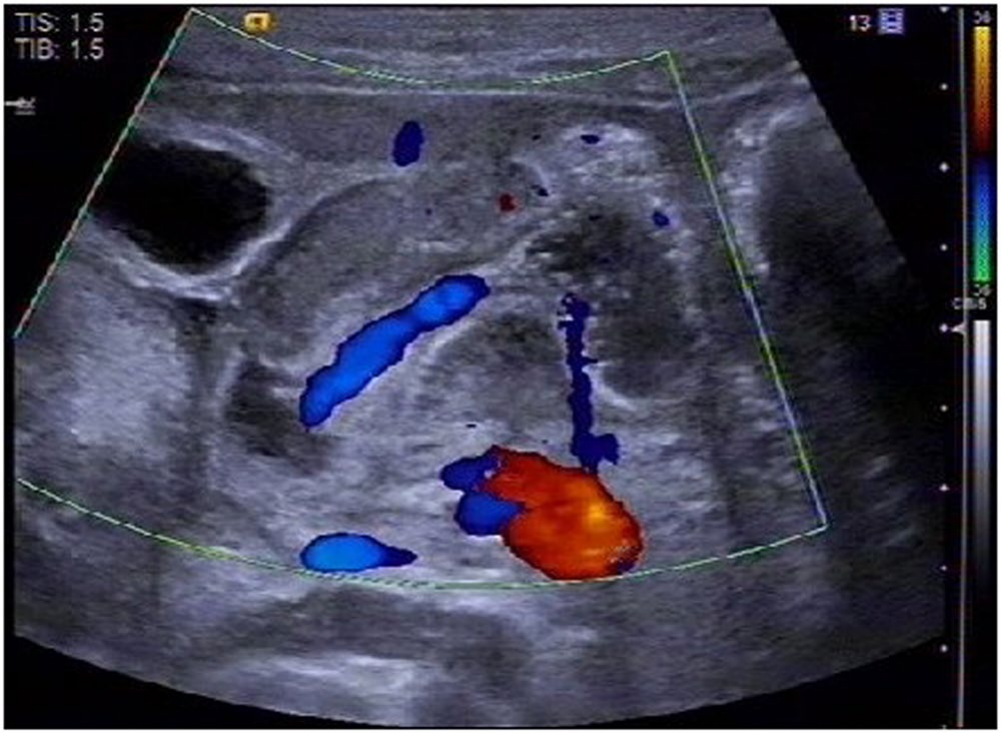
Doppler artifacts for examining the whole process of color blood flow passing through the Pylorus.

Contrast agent was injected through the stomach tube into the stomach to observe the flow condition in the pyloric canal (Fig. 2). By using color Doppler artifacts technique, the moving condition of contrast agent in the pyloric canal was observed clearly. When the liquid passed from the gastric antrum to the duodenum, color Doppler artifacts showed a mixed stack effect which was obvious and the signal was bright with a colorful flow signal (Fig. 3).

During 20 min observation by CDFI, 10 cases of 65 CHPS infants for the first through time was 5 min, 5–20 min in 25 cases, 30 cases had no obvious liquid through after 20 min. During the observation, liquid passing in 7 cases was observed for more than 3 times, 28 cases for 1–3 times. The mean pyloric diameter was about 1.98 ± 0.33 mm (Table 4).

**Table 4:** Comparison of liquid pass through the pylorus by contrast agent only and Doppler artifacts in CHPS group (n = 65).

Method	The first through time (min)		Times		Pyloric diameter (mm)
	<5	5–20	1–3	>3	
Contrast agent only	6	21	38	22	1.95 ± 0.35
Doppler artifacts	10	25	30	28	1.98 ± 0.33
t value					0.57
p value					0.47

## Discussion

The structure of pylorus tissue was similar to the gastric wall, from inside to outside, and was divided into four layers, mucosa, submucosa, muscular layer and placenta percreta. The inferior pylorus artery, which mainly originated from the gastroduodenum and right gastroepiploic artery and from their bifurcation, was the main region of blood supply to the pylorus^18^. Currently, ultrasound examination of CHPS are mostly in two-dimensional state of observation, few literatures reported the demonstration of blood flow distribution characteristics of hypertrophic pyloric tissues in the CHPS using CDFI^19^. In our study, the blood flow distribution characteristics of each layer of CHPS were observed clearly, which showed that the blood in the pyloric muscular layer was thick and was short rod arranged in parallel on longitudinally while it was radiating on the transverse section, and perpendicular to the pyloric diameter. The direction of blood flow in the serous layer and submucosal layer was a continuous strip and the pyloric tube diameter was parallel to the pyloric diameter on the longitudinal section, which provided some objective information clinically, which avoided the loss of larger blood vessels in surgery in order to reduce the blood loss and the risk of infection after operation. It is of great significance for surgical treatment. This study found that compared to the control group, the difference of blood flow grade in muscular layer and mucosal layer was significant. But in CHPS group, the difference of blood flow grade in muscular layer and mucosal layer was no statistical significance. However, we found that 31 cases with blood flow of pyloric submucosal distribution was grade II, 34 cases was grade III; 36 cases of pyloric muscular layer flow was grade II and 29 cases was grade III, which showed that the blood flow in the submucosal layer was more abundant than that in the muscular layer. Mucosal layer is composed of connective tissues, blood vessels and nerves, which is full of blood vessels, so blood flow is rich, which can be explained from the normal morphological structure and physiological function^20^.

Our study used color Doppler imaging to observe the condition of contrast agents through the pyloric canal. Together with the experience of domestic and foreign scholars^21, 22^, color Doppler artifacts can be broadly divided into the following categories: Firstly, there is no color or few color signals were observed in the area of blood flow. Secondly, too many color signals were observed in the blood flow. Thirdly, color signals appeared in the non-blood flow area. Fourthly, color signals or its shades of color change caused the misunderstanding in the direction and velocity of blood flow. In our study, the color signals appeared in the non-blood flow regions. The principle behind this is that it was similar to that of the “flame sign” caused by the spray of the ureteral orifice. It is an artifact that was generated by the rapid flow of liquid, which may be related to the presence of micro materials in the flow of liquid causing ultrasound scattering^23, 24^. In the planar ultrasonic condition, it is sometimes difficult to catch the movement of contrast agents through the pyloric canal because of the contrast echoes produced by the liquid as well as pyloric canal were not strong, which cannot reflect the pyloric stenosis objectively and may be misleading clinically. Using color Doppler artifacts, we can dynamically observe the whole process of liquid passing through the pyloric canal more intuitively, the time and frequency of liquid that passes into the stomach through the pyloric canal, and also measure the pyloric length, which in turn provides an objective basis for the clinical evaluation of hypertrophic pyloric stenosis.

Ultrasound examination was performed based on the subjective and objective factors. Subjective factors refer to the operator’s skill and experience, objective factors refers to the resolution and sensitivity of the blood flow by the equipment. In order to reduce or avoid the influence of the above factors, we tried to let the experienced physicians operate with higher grade ultrasound instrument, and the same person to check on the same instrument to reduce the error. In addition, because of the small study, the statistical results might show possible errors which were different between the two groups in age and gender, and may be considered as random which were not good enough.

In summary, ultrasound along with color Doppler imaging technology not only diagnose CHPS but also examine the distribution of blood flow in different mucosal layers and blood flow grade accurately. Also, examine if the contrast agent can pass through the pyloric canal successfully and pass through the condition. This provides an objective basis for the clinical evaluation of hypertrophic pyloric stenosis, also a selection for the treatment of infants, and provides clinical evidence to avoid rich blood vessels further extending its significance.

## Methods

### Study design and patients

Sixty-five infants were examined due to clinical suspicion of CHPS and were prospectively recruited in the study. From August 2006 to December 2015, 65 infants diagnosed with CHPS by ultrasonic and upper gastroenterography and confirmed by gastroscopy in the neonatal department of Guangzhou No.1 People’s Hospital, China and all the cases were in accordance with the diagnostic criteria of CHPS^25–27^ were enrolled in the study. At present, the more generally accepted CHPS ultrasound diagnostic criteria include the muscular layer thickness of at least 3mm and/or the length of the tube with at least 17mm^28–30^. All the cases had typical vomiting, stomach type and gastric peristalsis, abdominal palpable pyloric olive-shaped lumps were included, and the exclusion criteria were pylorospasm and hiatal hernia in children associated with vomiting. There were 59 males and 6 females, aged 15 to 103 days, weighing 2.2 to 6.6 kg in the CHPS group. The control group consisted of 50 infants (27 males and 23 females), aged 20 to 88 days, weighing 3.7 to 10.8 kg, hospitalized in the same period for other diseases such as pneumonia, urological problems or abdominal hernias, with no other clinical manifestations of pyloric stenosis. The presence of CHPS was confirmed by gastroscope; the absence of CHPS was determined by reviewing the medical records. The protocol for this study was approved by the Institutional Review Board of Guangzhou First People’s Hospital. All the patient’s family members provided written informed consent. All methods were performed in accordance with the relevant guidelines and regulations.

### Indicators and measurement

Ultrasonic instrument using Logiq company GE 9/E Vivid 9 ultrasound diagnostic instrument and Philips company IU22 ultrasound diagnostic instrument, the probe using high frequency linear array transducer and with a center frequency of 7.0~10.0 MHz and 7.0~12.0 MHz were used. Infants were fasted for 4 hours and gastric lavage was performed with tube reserve, by administering 10% chloral hydrate sedation (0.5 ml/kg). They are placed in supine position and scanning was performed in the longitudinal plane where the cardia and lower esophagus can be observed. Then the infant was turned towards left to observe the stomach cavity, stomach wall and the motility situation of the stomach. Then scanning was performed by turning the infant to the right, where the transverse of the pyloric canal can be found between the top of the right kidney and the lower of the gall bladder, turned at 90° to observe the long axis of the pyloric canal, the morphology and structure of the pylorus, and measure the thickness of the pyloric muscular, pylorus diameter and internal diameter, the length of the pyloric canal. The pylorus was then subsequently evaluated with color Doppler US. The presence of color signal in the muscular layer or mucosal layer was documented, and the number of signals (i.e. degree of flow) was classified into three-point scale^17^: Grade 1 meant no flow signal; grade 2 meant two to five signals (i.e. moderate flow); and grade 3 with extensive or continuous flow. Flow was confirmed with Doppler spectrum analysis. Then infuse the stomach with warm water or condensed milk as a comparison to the contrast agents, observed for 20 min, and then used the Doppler artifacts technique to examine the flowing condition of the contrast agents, and measure the diameter of pylorus.

### Statistics

Data was expressed using frequencies, percentages or means and standard deviation (SD). The t test was used to compare different parameters of the control and the CHPS groups, the blood flow of the muscular layer and the mucosal layer between the two groups, the condition of the liquid passed through the pyloric canal by using the contrast agent alone and combined with Doppler artifacts image technology in the CHPS group. A *P* value < 0.05 was defined as statistically significant. Statistical analysis was performed using SPSS 13.0 software package for windows (SPSS Inc, IL, USA). All data generated or analysed during this study are included in this published article.
